# Workflow‑Based Information Management Framework for Multicenter Research Studies: Design and Development

**DOI:** 10.2196/81119

**Published:** 2026-04-20

**Authors:** Hasan Sulaeman, Mars Stone, Roberta Bruhn, Karla Zurita, Anh Nguyen, Vincent Chiang, Jefferson Michael Jones, Xutao Deng, Brian Custer, Michael Busch, Eduard Grebe

**Affiliations:** 1 Vitalant Research Institute, 360 Spear Street, San Francisco, CA, 94115, United States, 1 (415) 923-5771; 2 Centers for Disease Control and Prevention, National Center for Immunization and Respiratory Diseases, Atlanta, GA, United States

**Keywords:** multicenter studies, large studies, open source, data system, data management, data infrastructure, ETL, extract transform load

## Abstract

**Background:**

Biological and health research is increasingly data-driven, with commercial and academic institutions generating data at unprecedented rates. The rapid pace of data generation, together with lessons learned during the COVID-19 pandemic, underscores the need for nimble, transparent, and dependable data infrastructures that enable rapid study execution and timely insights to inform public health policy and practice.

**Objective:**

This paper describes the workflow-based information management (WIM) framework, a flexible research information management system designed to support diverse epidemiologic workflows and data-intensive research projects.

**Methods:**

WIM was developed as a modular, workflow-oriented framework built on the open-source R (R Foundation) programming language and its extensive ecosystem of community-developed packages. The framework emphasizes reproducibility, adaptability, and transparency, enabling users to design and manage research workflows tailored to specific study requirements. We describe the architecture and core components of WIM and illustrate its application through representative epidemiologic research scenarios.

**Results:**

The framework supported high-volume, multiorganizational research; managing >3.7 million donation and testing records from 17 blood collection organizations across the United States. The WIM framework was readily adapted to a wide range of epidemiologic studies and research projects, demonstrating flexibility across varying data types, analytical needs, and operational contexts. By leveraging established R-based tools and workflows, WIM supported efficient data ingestion, processing, analysis, and reporting while promoting reproducible and collaborative research practices. The framework facilitated rapid iteration and reuse of workflows, addressing common challenges in managing complex and evolving research studies.

**Conclusions:**

WIM provides a flexible, open-source, and extensible approach to research information management for modern biological and health research. By integrating workflow-based design principles with the R ecosystem, the framework supports reproducible analysis, scalable research operations, and rapid study execution. WIM offers a practical solution for institutions seeking adaptable data infrastructure to support epidemiologic research and inform public health decision-making.

## Introduction

With technological advances in recent decades, bioinformatics and data management are becoming increasingly important to life sciences research [1-3]. Contemporary biological research is often dependent on the management, sharing, and analysis of large-scale, aggregated data, in particular for studies designed to tackle large scientific and societal issues such as the COVID-19 pandemic [4-6].

During the COVID-19 pandemic, scientific interest in specific public health questions shifted as the pandemic progressed [7]. For example, early seroprevalence studies were performed to determine the proportion of the population that had been infected with SARS-CoV-2. After the introduction of vaccines, these had to be modified to determine the proportion of the population that had been vaccinated, infected, or both [8-10]. In May 2020, the US Centers for Disease Control and Prevention (CDC), in partnership with Vitalant Research Institute (VRI), established the Nationwide Blood Donor Seroprevalence study (NBDS). In July 2020, NBDS launched the first phase of the study in collaboration with 17 blood collection organizations across the United States and Puerto Rico. Multiple testing laboratories, including VRI and Creative Testing Solutions, captured, tested, and analyzed approximately 150,000 blood donation specimens monthly in a serial cross-sectional seroprevalence survey. As the pandemic evolved, a large proportion of the population had infection-induced antibodies or had been vaccinated. To determine if infections occurred before or after vaccination or to detect reinfection, longitudinal data is required. The need to detect reinfections and infections in vaccinated individuals led to the launch at the start of 2022 of the second phase of the program: the Nationwide Blood Donor Cohort study (NBDC). The NBDC program switched from a cross-sectional to a longitudinal study format to follow a cohort of blood donors from BCOs to address questions such as the incidence of infection (in vaccinated and unvaccinated individuals) and multiple sequential infections (reinfections) with SARS-CoV-2, waning antibody titers following vaccination or infections, and correlates of protection against SARS-CoV-2 infection [11,12]. Given the fluid nature of these large studies, a secure and nimble data infrastructure was needed to meet the studies’ needs, including management of large quantities of data on donors and donations from BCO records, serologic testing data, and responses to electronic donor surveys.

BCOs are primed for nationwide studies such as the NBDS and NBDC, with a physical infrastructure already in place to collect and test biospecimens from blood donations, including capturing residual specimens after routine blood screening for additional research testing and executing electronic surveys of participating donors. However, the data management framework and infrastructure to facilitate research programs built on the blood collection system would benefit from further development, a challenge many other organizations face and are attempting to tackle [13,14]. Due to the volume and complexity of the NBDS and NBDC data, traditional methods of data manipulation and management using software such as spreadsheet-based software were not an option. Though there are numerous commercially available data and project management software systems, their use would require a costly upfront license fee and/or a monthly cost incurred from services rendered for a software-as-a-service platform. Furthermore, off-the-shelf solutions typically require extensive customization to meet the needs of large and complex research programs.

Fortunato and Galassi (2021) [15] defined open-source software as “any computer program released under a license that grants users rights to run the program for any purpose, to study it, to modify it, and to redistribute it in original or modified form.” R and Python (Python Software Foundation) are 2 popular data manipulation and analysis programming languages that fall into the category of open-source software. We chose to build our data management infrastructure in R due to its popularity among life science professionals, versatility, low barrier to entry, and a strong community of developers that has built an ecosystem of data-related packages [16,17]. Using the same principles as the framework we detail here, an analogous data system could easily be built in Python or any other open-source general programming language.

The design of our workflow-based information management (hereafter WIM) framework follows a few distinct design principles to achieve the goal of a nimble and reusable informatics management system that could reliably handle large amounts of data using open-source programming languages. First, our framework follows the FAIR (Findable, Accessible, Interoperable, Reusable) principles detailed in Wilkinson et al (2016) [18] for data reusability, accessibility, and system interoperability. Second, our framework follows the design principle of loose coupling, wherein components are only weakly associated with each other or the system, and so changes in 1 component least affect the performance of other components [19]. This principle allows the framework to be agnostic regarding operating systems and underlying platforms such as the relational database management system. Lastly, due to the fluidity of research studies, to prioritize reusability and adaptability of the framework, we opted not to have a formal component model [20].

## Methods

### Concept

The framework is built on modules, some automated, used in the same way functions from packages are used in R, and is publicly accessible on GitHub (GitHub, Inc) [21]. The modules are called and managed using the *box* package, which negates the need to import each function or to publish the suite of modules as an R package [22]. Additionally, all settings used by the modules are set using a single project-wide configuration file in the yet another markup language (YAML) format, which includes information on quality control (QC) processes, data dictionaries used by the study, credentials where required to run certain processes, database connection strings, and any other pertinent information used in data ingestion and reporting [23]. The use of a configuration file streamlines the implementation of changes in data flow, formatting, and reporting and lessens the time required to apply any changes to the data system, while also allowing multiple data managers to execute the same functions and scripts with any individual configurations managed in a separate configuration file.

The modules are platform-agnostic and can be run on either a cloud platform or locally with little to no input, depending on how much of the process requires manual review. They could also be run on a set schedule as a *cron job*, a system for scheduling specific tasks on Unix-based operating systems (eg, Linux [DragonByte Technologies] and macOS [Apple Inc]), or automatically when required using a listener to respond to an external action. In our case, for pragmatic reasons, the modules are run locally on desktop machines by one or more administrators. Separately, though modules run processes through R, an external connection is required to run certain processes, such as performing data retrieval from an external source, monitoring a secure file transfer protocol (sFTP) server for new data deliveries, writing processed and quality-controlled data to a central database, and transferring data to other organizations. These connections are made in R using certain packages, including *curl*, *RCurl*, and *sFTP* [24-26].

Lastly, a central goal in the implementation is for all relevant stakeholders to have access to both the managed central database and the version-controlled code repository, managed using Git. To lower the technical skill floor required for accessing this study’s database, a custom front-end was built using Python and Flask (Pallets), which caters specifically to the organization and stakeholders’ data needs and is abstracted. The Git repository for the modules includes all the information and scripts required to install and run the modules, with users having only to install the required dependencies using a script included in the Git repository.

### Data Sources and Types

**Figure 1.**
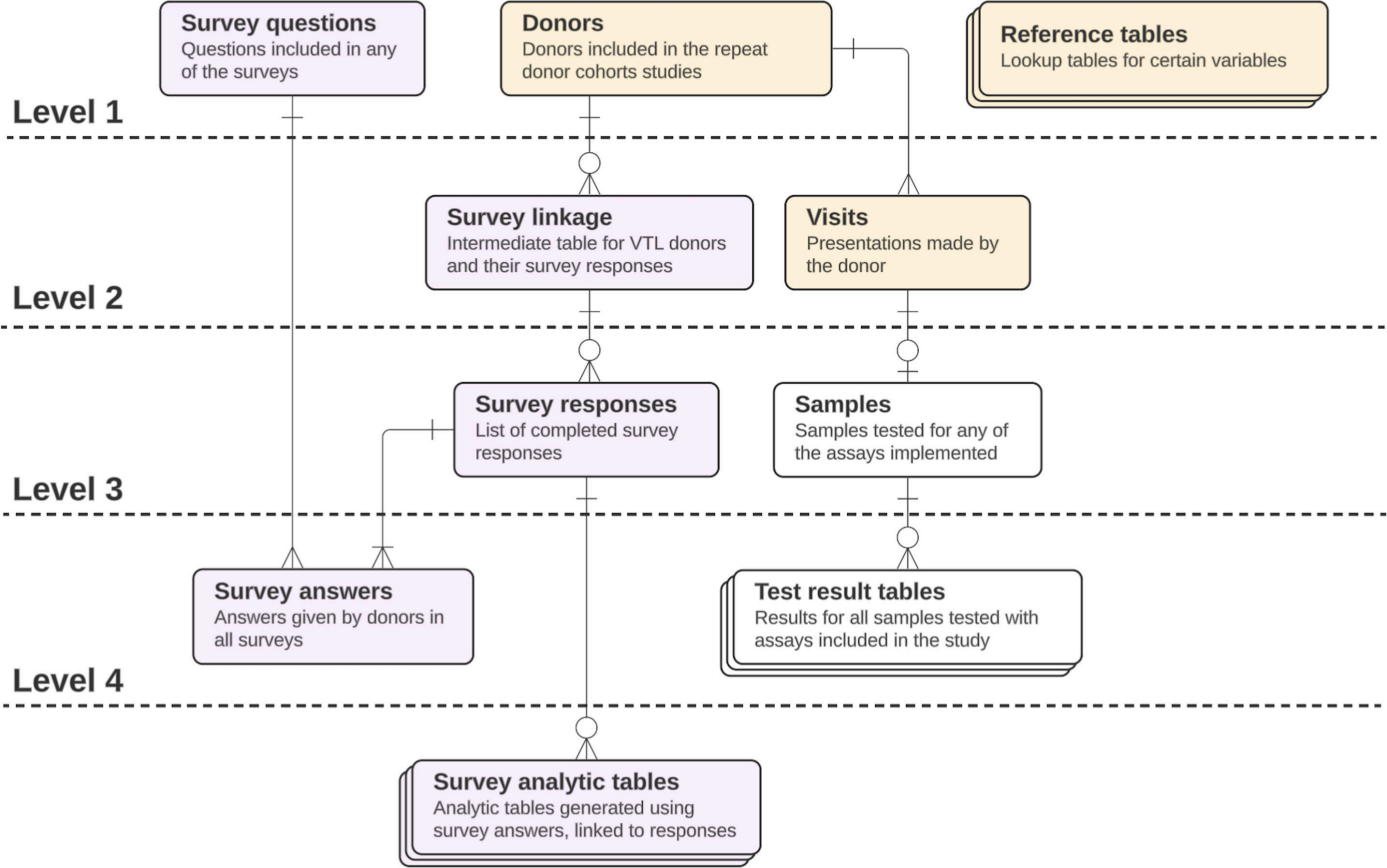
Simplified ERD for the repeat donor cohorts program. Levels are used to imply dependencies between the different tables. Purple, yellow, and white are used to denote survey data, donation data, and testing data, respectively. Standard cardinality notation is used. ERD: entity relationship diagram.

Data sources for the NBDS and NBDC included donor, donation, and blood specimen testing data (Figure 1). When the NBDC study launched, donor electronic survey data were added as a data source [27,28]. Donor and donation data for the program are extracted from BCO operational data systems. Donor data includes time-invariant donor characteristics such as birth date and blood group, while donation data includes information collected at each donation event, for example, the blood collection procedure, responses to routine questionnaires administered at the time of donation, as well as other data points that might differ for a given donor over time. Data points such as ZIP code of residence, sex, race, and ethnicity were reported by the donor at the time of donation. It is worth noting that reference tables, also known as lookup tables, were generated and used for tracking changes to donor identifiers and certain donation-level data where the original data point from the source should be kept, along with any study-specific interpretation we applied. For example, the way organizations record and group donor race and ethnicity data might differ from how a study groups these data. By implementing a lookup table that links the original data to the study-specific data grouping, stakeholders can revise groupings, if necessary. Testing data includes all valid serological test results from assays in the program, whether for routine study-directed testing or for assay validation substudies [29,30]. Specifications for which variables are captured, stored, and reported differ between assays and are dependent on the assay manufacturer’s instructions for use and determinations of the most relevant measurements.

Lastly, donor survey data for the NBDC can be split into 2 other data types: survey question information and survey response information—both of which are equally important to properly manage. Survey questions for the program often change between different survey rounds to accommodate the changing needs of this study, so there is a need to manage question information while tracking question equivalency across survey rounds. Due to survey questions sometimes changing, survey responses, defined as a completed survey form, must be managed separately from the discrete answers to a question. This is so that answers to equivalent questions across survey rounds can be parsed and analyzed without requiring excessive processing time or manual recoding during analysis.

### Data Dictionary

The NBDS program accepted frequent data submissions from 17 BCOs and multiple testing laboratories across the United States and Puerto Rico. The NBDC program, on the other hand, while only accepting submissions from 2 organizations and 2 testing laboratories, expanded the scope and quantity of data collected, stored, and managed for its cohorts. Both studies required comprehensive data dictionaries, regularly updated to reflect changes made to study procedures and methods, and detailing how to format data for each type of data transfer between organizations, including what values or valid ranges are acceptable for each field. Data dictionaries for both the NBDS and NBDC programs are included in MultimediaAppendices 1  2.

### Data Flow

**Figure 2.**
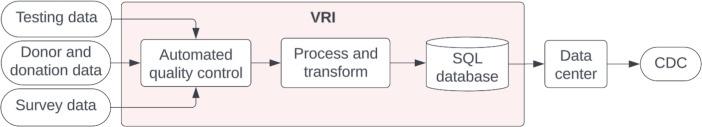
Workflows defined by the framework. Workflows include an extract, transform, load process from each data source, a process to manage data while at rest, and a process for reporting to the data center and later the US Centers for Disease Control and Prevention. CDC: US Centers for Disease Control and Prevention; VRI: Vitalant Research Institute.

Though the data flow for the NBDS and NBDC programs differed slightly due to different data sources, types, and testing algorithms applied to biospecimens, the general flow of data remained the same (Figure 2). Data from all sources first flowed to VRI, where it underwent QC and, if the submission passed quality control procedures, was imported to this study’s database. The data submission was then reported to the data coordinating center (DCC), the contract research organization Westat, where it underwent a second QC step, where primary analysis took place, and was finally transferred to the sponsor (CDC).

For the NBDS, to accommodate varying testing and sample flows, participating BCOs were binned into groups, each of which had a distinct data transmission format and schedule [12]. Groups evolved with the program and adapted to changes in the testing algorithm and the capacity of testing laboratories. For the NBDC, data flow differed by data source and organization. Donor and donation data for Vitalant flowed to VRI, while for ARC, the submission went directly to the data broker. Testing results are always routed through VRI before going to the DCC, while survey results were reported directly to the DCC for both Vitalant and ARC (Figure 2)

### Study-Specific Relational Database

The use of a study-specific database was critical in the day-to-day operations of both the framework and this study itself (Figure 2). Having certain restrictions inherent in the structure of the relational database, which included length of the field, primary and foreign keys, data types, allowable potentially identifying information, and allowable codes for categorical fields (enumerated types), establishes this study’s database both as the canonical source of study data and as a redundant QC process by passively ensuring data ingested is in accordance with this study’s data dictionary as well as all relevant regulations surrounding human-participants research and privacy protections. In our case, with the hierarchical structure of donor, visit, testing, and survey data, a study database was instrumental in keeping data integrity across tables. Lastly, it is important to note that for organizations working with potentially identifying information, such as hospital records, granular data governance is crucial to augment privacy protections by allowing only relevant stakeholders to view certain parts of the database.

### Automated Secure Data Retrieval

This study relied on 2 data transfer methods that are encrypted both in transit and at rest: Microsoft OneDrive (Microsoft Corp) and an sFTP server. The module responsible for automated secure data retrieval performs several processes and uses Microsoft Workflows (previously Microsoft Power Automate; Microsoft Corp) as a method of notifying submitters and stakeholders of any new submissions. The workflow or module of the framework is also responsible for notifying stakeholders whether the submission passed QC and was accepted, or did not pass the QC checks and was rejected. Each participating organization is given a separate directory (also known as a bucket) on the sFTP server, along with credentials for their account. Within each bucket, 3 directories are made: upload, download, and archive. Users are only allowed to add or remove files in the upload and download directories, where they can upload data submissions or download data validation reports and rejected data submissions, respectively. The archive folder is where accepted submissions are moved and stored as a backup and for data audit purposes.

Automated listeners monitor each directory, with a process that is triggered when a file is altered or added in the directory. A change in the upload directory triggers a new submission email to all relevant stakeholders, while a change in the download and archive directories triggers rejection and acceptance emails, respectively. When a new submission is added to the upload folder, an R module downloads the file and removes it from the upload directory. The module then performs a QC process specified in the configuration file and, depending on the outcome of the quality control check, performs 1 of 2 actions: if the submission failed QC, the module uploads an itemized list of all quality control issues encountered to the download folder, which prompts a submission rejection email to all stakeholders with the itemized list of quality control issues encountered attached. Data type, length, date, and enumerator checks are examples of what the quality control process entails. If the submission passes quality control, the accepted submission is uploaded to the archive folder, the data is imported into this study’s database, and a submission acceptance email is sent out to all stakeholders.

### Data Intake Workflows

#### Donor and Donation Data

The extract, transform, load (ETL) process for donor and donation data differed between the NBDS and NBDC programs, as well as by organization for both studies. For the NBDS, donation data was transferred from all participating organizations through the sFTP server, while for the NBDC, the data for Vitalant was transferred through Samba servers to VRI. Donor and donation data for ARC were sent directly to the DCC. For the NBDS, all submissions were required to adhere to the agreed-upon data dictionary and were submitted in accordance with a data submission guideline document shared with all participating organizations (Multimedia Appendix 3). For the NBDC, the only processes required were QC and data transformation for Vitalant donor and donation data. Once Vitalant’s data submission passed quality control and was transformed per this study’s data dictionary, the data were imported to the study database (Multimedia Appendix 2).

#### Laboratory Testing Data

Testing data for both the NBDS and NBDC programs were generated by testing and immunology laboratories, with each testing laboratory having a separate workflow and R module. Transmission of test results from the test laboratory was sent via a cloud service, and a QC check was performed to ensure adherence to this study’s data dictionary. For testing data from the immunology laboratory, output from testing instruments was retrieved, reformatted to fit this study’s data dictionary specifications, and imported into this study’s database automatically by an R module developed for this purpose (Figure 2).

#### Donor Survey Data

**Figure 3.**
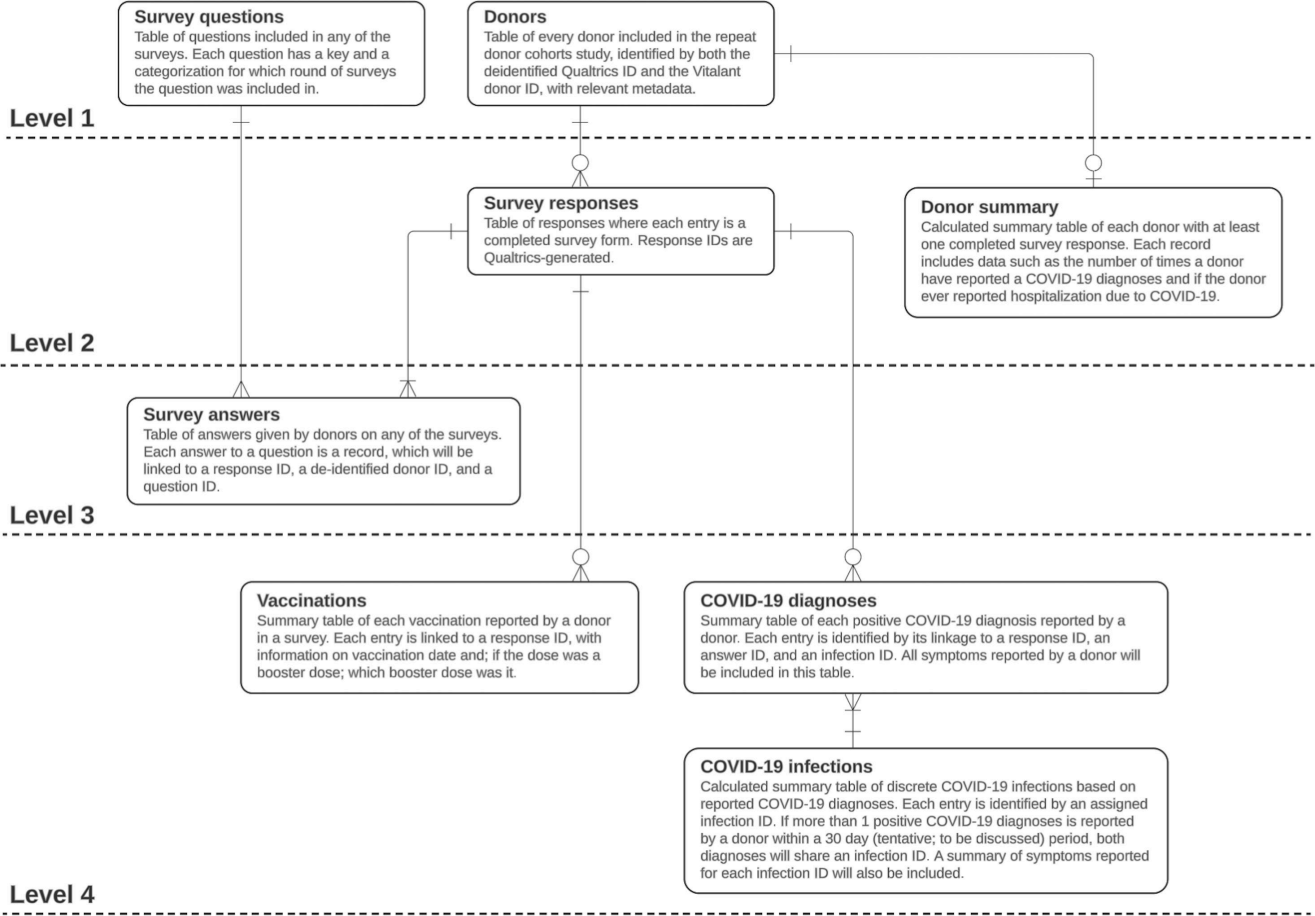
Management of survey data in this study’s database. Levels are used to imply the linkage dependency of tables. Level 1 tables do not need primary and foreign keys to link to another table, while levels 2, 3, and 4 rely on linkage with subsequent levels. Standard cardinality notation is used.

Donor survey data consisted of both survey questions and responses over time. As survey questions change to accommodate changes to this study over time, the data management approach must track how these questions shift and change over time—maintaining equivalency of questions across survey rounds. The data management approach also must be able to record answers to each question without having to change the database table structure with every change in survey question (Figure 3). It was also important to capture analysis-ready data that integrated data captured across survey rounds in a consistent format.

The database structure for the donor survey data was generally categorized as either a raw data table or an analytic table. The raw data tables consist of survey questions, survey responses, and survey answers, while analytic tables include any tables using the survey data to derive information that might be beneficial for analysts without having to parse the raw answers. The survey questions table records every question ever given to donors across all survey rounds and assigns to each question a question ID. Equivalent questions across rounds share the same question ID for ease of analysis. The survey responses table records whether a respondent has a completed response for each survey round and whether the respondent consented for their answers to be used in research (ie, 1 row per respondent per survey round). For donors who did not consent for their answers to be used in research, none of their survey answers are recorded in the database, though they are still recorded as having responded to the survey and flagged as nonconsenting. While respondents generally did not answer any further questions if they failed to provide consent at the outset, consent could be withdrawn later in the process, and these technical safeguards ensured that no responses were recorded without consent to use the responses in research. The survey answers table consists of several fields, including a unique response ID that links to the responses table, a question ID that links to the survey questions table, and the answer given to the specific question by the respondent on the survey.

Two modules are responsible for managing the survey data, both of which rely on raw output from the survey platform. The first module is responsible for assigning equivalency to each question based on the question ID and for updating the survey questions table, while the other module parses survey responses and updates both the survey responses and survey answers table.

### Reporting Workflows

Reporting is done through a module for each type of report defined by the program. For example, reporting for the NBDS program only required testing reports. In contrast, the NBDC program required donor, donation, survey, and testing reports. While each reporting module pulls information from the configuration file on which fields to extract from which tables in the database, and how to transform the data to fit the data dictionary’s reporting specifications, extra code is generally needed for specific requirements that require more specific QC steps than the standard checks for data format and length. This necessitates more modular code to be included. For example, changes in how a laboratory test is configured might require changes in the quality control process, for example, to check for values in other fields, or to transform values based on one or more entries in other fields.

### Ethical Considerations

All blood donors consented to the use of deidentified, residual specimens for further research purposes. Consistent with the policies and guidance of the University of California, San Francisco Institutional Review Board, VRI self-certified the use of deidentified donations in this study as not meeting the criteria for human participant research. CDC investigators reviewed and relied on this determination as consistent with applicable federal law and CDC policy (45 C.F.R. part 46, 21 C.F.R. part 56; 42 U.S.C. § 241[d]; 5 U.S.C. § 552a; 44 U.S.C. § 3501). The study number is Pro00056783. The donor surveys conducted by Vitalant were conducted under a protocol supervised and approved by the Advarra Institutional Review Board (Pro00056783) and linked to biospecimens in deidentified form.

## Results

### Customizability

The framework we developed resulted in a data system that is robust, reusable, and adaptable. Several factors contribute to the adaptability and reusability of the system. First, by separating generic code from the study-specific information, the system can adapt to changes in the study or be reused for another study with a greatly reduced investment of time to set up a study-specific data system (Table 1). The use of a configuration file that instructs the base code on how to access certain study-specific resources; what data types, variable lengths, and data types are allowed for specific entries; and how to generate multiple types of reporting required by the study or project allows for the study’s data system to be quickly adapted to changes in the study or implementation of new substudies, without lengthy development time and making extensive changes to the code. An example would be how changes to a study’s codebook are implemented. For systems where certain fields or information are hard-coded, implementing a change means changing the base code. In the case of our system, editing the configuration file is sufficient to implement the changes.

**Table 1.** Overview of the key design principles that guided the development of the WIM^a^ data management framework.

Principle	What the principle seeks	WIM design features	Examples from NBDS^b^/NBDC^c^
Findable	Data and metadata can be easily found via clear documentation and identifiers	Comprehensive data dictionaries and persistent identifiers are enforced across different studies	Shared IDs kept consistent across NBDS and NBDC to enable cross-study linkage and downstream analyses
Accessible	Users can retrieve data with appropriate authorization and governance	Abstracted database web frontend with authentication and granular access control, lowering the skill floor while supporting governance	Nontechnical collaborators access curated tables and reports through the web frontend, with role-based permissions
Interoperable	Systems, tools, and data can work across platforms and contexts	Platform-agnostic modules can connect to multiple software platforms; deployable on-prem or cloud	ETL^d^/QC^e^ modules run on Linux or Windows (Microsoft Corp) and interface with different database engines and file exchange endpoints,
Reusable	Data and tools are packaged to maximize reuse across studies and time	Single project-wide YAML^f^ configuration externalizes study-specific logic (ie, dictionaries and QC rules) from core code. Omission of a formal component model to preserve flexibility	Reused ingestion, QC, and reporting modules between NBDS and NBDC with primarily configuration changes, minimizing code edits and reducing setup time
Loose coupling	Components change with minimal ripple effects on others	Strict modularization (ingestion, QC, ETL, and reporting separated). Configuration- driven behavior	Updating a QC rule or adding a new data source did not require edits to ingestion listeners or reporting modules

a WIM: workflow-based information management.

b NBDS: Nationwide Blood Donor Seroprevalence.

c NBDC: Nationwide Blood Donor Cohort.

d ETL: extract, transform, load.

e QC: quality control.

f YAML: yet another markup language.

Second, for data with significant structural variability over time, such as the survey data in our case, native functionality of database engines should be leveraged (Figure 3). For example, survey data for our study changed regularly between survey rounds, and keeping the data in wide format would mean having to manage a very wide table. As a wide table would mean having to change table configurations and column names as changes are implemented to the surveys, we elected to store survey data in a long format—with discrete tables for survey questions, responses, and answers. The use of long format for both survey answers and questions negated the need for tables with a column for each question ever asked on the survey. Additionally, having both the survey questions and answers in a long-format table means that the general data structure can be preserved, even if changes are made to the survey questions, and that the survey questions table can be used as a reference table to check for equivalency of specific questions between different rounds of the survey.

The last factor to contribute to the framework’s reusability and adaptability is being flexible with respect to connections with other systems, along with loose coupling. Interoperability, as defined by Wilkinson et al (2016) [18], is the ability of data or tools from noncooperating resources to integrate or work together with minimal effort. By using and interoperating with other preexisting data systems and platforms, including proprietary software systems, the framework can fill in gaps in functionality and be implemented in parallel with both legacy and new systems.

### Evaluation of Use Case

We used deployment efficiency, adaptability, and update latency, data throughput, and reusability of core modules as key operational indicators used to evaluate the framework. These metrics reflect real-world performance under high-volume, multiorganizational research conditions. Taken together, these metrics highlight WIM’s ability to support large, complex, and rapidly evolving research programs by providing a scalable, adaptable, and reusable data-management infrastructure.

### Data Throughput and QC Performance

**Figure 4.**
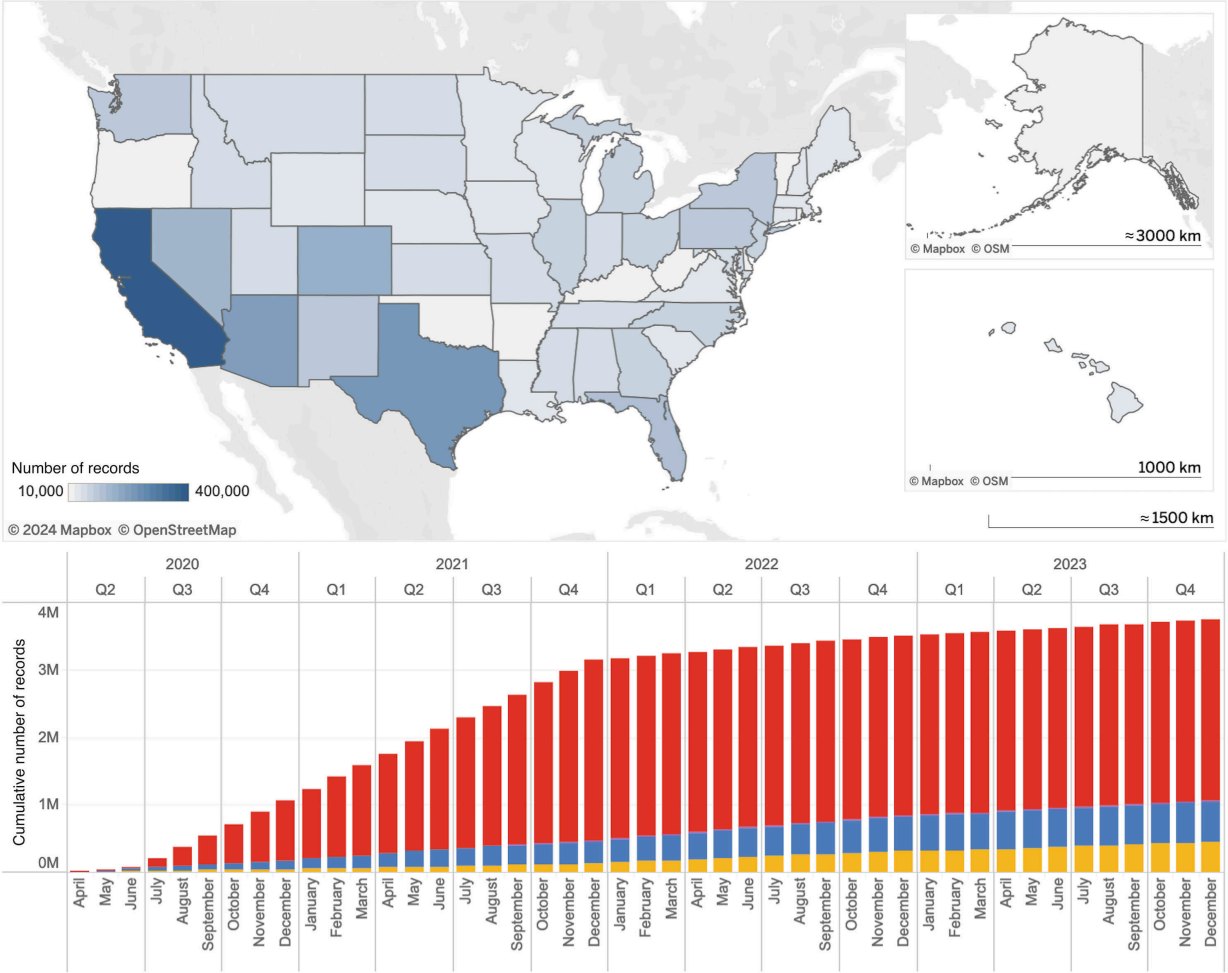
Number of records collected and managed by the NBDS and NBDC programs. (A) Number of records collected by state, with 270,319 records excluded due to missing geographic information. (B) Number of records accumulated over the two programs’ lifetime. Colors yellow, blue, and red denote whether the record was collected for the repeat donor cohorts 2023, Vitalant repeat donor cohorts 2022, or the National Blood Donor Serosurveillance study, respectively. NBDC: Nationwide Blood Donor Cohort; NBDS: Nationwide Blood Donor Seroprevalence.

For the NBDS study, our study data infrastructure handled 2,670,225 donation and testing records from all 50 states in the United States over the lifetime of this study (Figure 4). These records were generated by 17 BCOs and were submitted to VRI monthly. For the NBDC study, our study data infrastructure handled a total of 1,064,381 donation records from 65,524 donors who were longitudinally followed over the course of 2 or more years (Figure 4). Donation and donor data were generated and updated on a quarterly basis, while testing data were updated on a weekly basis.

### Deployment Efficiency

For deployment efficiency, initial system deployment required approximately 6 months for the NBDS program, whereas deployment for the more complex NBDC program required just over 2 months. This reduction in setup time reflects the reusability of core modules and the ability to externalize all study-specific logic in the YAML configuration file, allowing new implementations to leverage an existing codebase with minimal modification. For other studies with different data needs, only study-specific logic and QC checks would have to be developed or adapted before implementation.

### Adaptability and Update Latency

Operational and methodological changes to this study, such as updates to data dictionaries, QC criteria, reporting formats, or survey structures, can be implemented typically within 1 day for minor revisions and within a few days for more complex changes involving changes to multiple workflows or a drastic overhaul of study design. As WIM isolates study-specific rules from core modules and uses an external configuration file, updates could be integrated rapidly into ETL, QC, or reporting pipelines with little to no change to the underlying software.

The framework successfully supported very high data volumes, which include 2.67 million donation and testing records in NBDS and over 1 million longitudinal donation records in NBDC. Data ingestion and reporting vary in size and complexity, but the framework handled data ingestion and reporting tasks of up to 500,000 rows seamlessly. Automated QC workflows processed each data submission immediately upon arrival, applying rule-based checks for field formats, enumerated values, date validity, and cross-table integrity. The use of automated listeners and rule-based validation eliminated the need for manual prescreening and ensured rapid feedback to submitting organizations.

### Reusability of Core Modules

For the NBDS and NBDC studies highlighted here, processes such as ETL routines, QC, secure data retrieval, and reporting were reused with no modification. Study-specific customization was largely restricted to configuration files, data dictionaries, and survey-specific logic. This high degree of functional reuse demonstrates the portability of the framework and supports efficient deployment in new research settings.

## Discussion

### Overview

Modern data generation techniques in life science research increasingly result in large quantities of data. With the increasing rate of data generation comes an increasing need for reliable, stable, and sophisticated information management systems to manage, store, and share data [2,31,32]. When large datasets from multiple, often disjointed, data sources can be accommodated, our ability to efficiently study large societal and scientific challenges is greatly increased (eg, studying population-wide effects of the COVID-19 pandemic) [33-35]. In this paper, we describe how our approach of developing a modular, flexible, and interoperable data infrastructure using open-source tools allowed us to nimbly manage data in rapidly evolving nationwide COVID-19 studies during the pandemic, and how we can scale, reuse, and adapt the same framework to manage other large multicenter studies with minimal changes to the core software.

Despite the availability of both commercial and open-source data-management platforms, the WIM framework provides several contributions that differentiate it from existing systems and were essential to supporting the rapidly evolving NBDS and NBDC research programs. First, it uses a single project-wide YAML configuration file to control all modules responsible for data ingestion, transformation, QC, reporting, and database interactions. This design places study-specific logic entirely outside the core codebase, allowing substantial changes to data dictionaries, quality control rules, or reporting specifications to be implemented without modifying the underlying software. This approach reduces development time, lowers operational complexity, and enables modules to be reused with minimal reconfiguration across multiple studies. Second, WIM achieves a high level of modularization and loose coupling, in which each workflow module operates independently. This stands in contrast to many proprietary systems that require vendor-managed customization or open-source pipelines that integrate ingestion, processing, and reporting in a rigid or monolithic structure. By separating generic functions from study-specific rules, WIM supports rapid iteration as research needs evolve and minimizes unintended downstream effects when updating a single module.

WIM’s architecture is designed for portability and reusability beyond the described COVID-19 serosurveillance programs described. Core modules for secure data retrieval, ETL processes, QC, configuration management, and reporting can be repurposed for new studies with only changes to configuration files and study-specific dictionaries. This level of reuse allowed implementation time for NBDC to be reduced from approximately 6 months for NBDS to just over 2 months, even as the complexity and data sources increased. The combination of configuration-driven adaptability, rigorous modularization, automated QC workflows, and open-source extensibility makes the framework a flexible and scalable solution for large, complex, and multiorganizational research environments.

In building the framework and the data system implemented using the framework, several design principles were followed with the goal of a nimble and reusable system that could be sustainably used in a myriad of different studies and implementation environments with minimal modification. The FAIR principles are detailed in Wilkinson et al (2016) [18], the principle of loose coupling, and the omission of a formal component model are the principles that allowed us to develop our framework with sufficient flexibility [19,20]. These design principles allow for a sustainable implementation, as the only changes and maintenance required on the system are dictated by updates to the open-source components used by the system.

The FAIR principles for data management suggest that contemporary data resources, tools, vocabularies, and infrastructures should exhibit the qualities required to encourage discovery and reuse [18]. Our framework follows these design principles in multiple ways. First, for the data to be findable, we keep documentation in the form of data dictionaries and enforce a persistent identifier across multiple studies and data systems for similar data types (eg, primary identifier for a blood donation is the same across studies). Second, for the data to be accessible, we built and manage an abstracted database frontend web application as part of the framework to lower the skill floor of stakeholder access to the data. Through user authentication and granular access control, the use of a web-based front-end application also contributes to data governance [13]. Third, for the data system to be interoperable, we have designed our data system to be agnostic with respect to operating systems and database engines, and to be able to connect to multiple other software platforms. Lastly, for the data to be reusable, we prioritized bidirectional compatibility and stability, wherein the data collected and managed in any 1 study should be linkable to and available for use in analyses of future data, as appropriate [20], while complying with applicable ethical and IRB requirements.

Two further guiding design principles we followed were those of loose coupling and the omission of a formal component model from the framework [19]. The omission of a formal component model was motivated by the fact that research is often not a linear endeavor, with changes to the process happening regularly as priorities shift. This is especially true for our work on the NBDS and NBDC programs. For this reason, modules were separated through workflows: data ingestion, management, and reporting. Each of these workflows can have multiple components, depending on the complexity of the study, but each workflow maintains its function, and modular code can be added or removed depending on the need.

On loose coupling, we took inspiration from ethology and decided that our framework should be agnostic with respect to operating system, relational database system of choice, and deployment method [20,36]. By choosing to develop our data system in R, and by omitting any functions that are dependent on the operating system, we allowed for the code to be run on any operating system that could run R. This also means that the system could be deployed on on-premises or cloud infrastructure. In our case, on-premises deployment was chosen, but in future studies, we plan to make use of cloud deployments, depending on cost, data security, and cross-organization access requirements.

While adaptability and data throughput capacity are important in any large-scale research data infrastructure, for human participants’ research we consider data integrity and the protection of sensitive personal information of participants to be of equal importance. Data integrity for the system is maintained through appropriate QC steps at every stage of data processing, along with the appropriate redundancies. For example, a data submission might be put through 2 QC steps, in parallel or sequentially. Automated or semiautomated handling of data submissions further supports data integrity, since rejected submissions that failed QC checks are sent back to submitters, along with a report providing detailed information on quality problems, for review, correction, and resubmission. For the protection of sensitive personal information, steps are taken to minimize the risk of a data breach by encrypting data in transit and at rest, making use of appropriately managed infrastructure (including vulnerability monitoring and an appropriate patching schedule), while also minimizing the collection and storage of personally identifying information. This ensures that even in the event of a data breach, the risk to donors or participants is minimized. An example of a practice we used to minimize the identifiability of research participants was maintaining dates of birth only at the month level and avoiding storage of multiple indirect identifiers. Further examples may include storing geographic information in formats that encode larger areas, such as 3-digit rather than 5-digit ZIP codes.

### Conclusions

With life sciences research becoming increasingly reliant on large, interconnected datasets and databases, the need for adaptable, reusable, and scalable methods to manage and curate data at an organizational or multiorganizational level will also increase [14,37]. Our work shows that open-source software, along with community-led software ecosystems, can be used to meet this need and provide superior functionality and flexibility to costly proprietary data management platforms.

With the rapid advancements in technology, bioinformatics and data management have become crucial to life sciences research. This is especially true for large-scale studies addressing significant scientific and societal issues, such as the COVID-19 pandemic. The described framework was built on the need for a nimble and abstracted data management system for multicenter studies. This was achieved by using modular components, and a single project-wide configuration file in YAML format sets all module settings, including QC processes, data dictionaries, credentials, and database connection strings. These features streamline data quality control, data flow, and data formatting, while lowering the effort required to implement changes and lowering the skill floor required to manage a system that usually requires a skilled data manager.

## Supplementary material

10.2196/81119 Multimedia Appendix 1 Data dictionary used for the first iteration of this study.

10.2196/81119 Multimedia Appendix 2 Data dictionary used for the repeat donor cohort iteration of the serosurveillance study.

10.2196/81119 Multimedia Appendix 3 Data submission guidelines used to train collaborators during the data submission process.

## Abbreviations

CDC: Centers for Disease Control and Prevention
DCC: data coordinating center
ETL: extract, transform, load
FAIR: Findable, Accessible, Interoperable, Reusable
NBDC: Nationwide Blood Donor Cohort
NBDS: Nationwide Blood Donor Seroprevalence
QC: quality control
sFTP: secure file transfer protocol
VRI: Vitalant Research Institute
WIM: workflow-based information management
YAML: yet another markup language

## References

[1] Pal S, Mondal S, Das G, Khatua S, Ghosh Z (2020). Big data in biology: the hope and present-day challenges in it. Gene Rep.

[2] Iqbal N, Kumar P (2023). From data science to bioscience: emerging era of bioinformatics applications, tools and challenges. Procedia Comput Sci.

[3] Liu Y, Chen Y, Han L (2023). Bioinformatics: advancing biomedical discovery and innovation in the era of big data and artificial intelligence. TIME.

[4] Boles NC, Stone T, Bergeron C, Kiehl TR (2017). Big data access and infrastructure for modern biology: case studies in data repository utility. Ann N Y Acad Sci.

[5] Attwood TK, Blackford S, Brazas MD, Davies A, Schneider MV (2019). A global perspective on evolving bioinformatics and data science training needs. Brief Bioinformatics.

[6] Dash S, Shakyawar SK, Sharma M, Kaushik S (2019). Big data in healthcare: management, analysis and future prospects. J Big Data.

[7] Stone M, Di Germanio C, Wright DJ (2022). Use of US blood donors for national serosurveillance of severe acute respiratory syndrome coronavirus 2 antibodies: basis for an expanded national donor serosurveillance program. Clin Infect Dis.

[8] Adalja AA, Toner E, Inglesby TV (2020). Priorities for the US health community responding to COVID-19. JAMA.

[9] Rahman S, Rahman MM, Miah M (2022). COVID-19 reinfections among naturally infected and vaccinated individuals. Sci Rep.

[10] Cohen C, Pulliam J (2023). COVID-19 infection, reinfection, and the transition to endemicity. Lancet.

[11] Jones JM, Manrique IM, Stone MS (2023). Estimates of SARS-CoV-2 seroprevalence and incidence of primary SARS-CoV-2 infections among blood donors, by COVID-19 vaccination status - United States, April 2021-September 2022. MMWR Morb Mortal Wkly Rep.

[12] Fink RV, Fisher L, Sulaeman H (2022). How do we…form and coordinate a national serosurvey of SARS-CoV-2 within the blood collection industry?. Transfusion.

[13] Leonelli S (2013). Global data for local science: assessing the scale of data infrastructures in biological and biomedical research. Biosocieties.

[14] Harrow J, Drysdale R, Smith A, Repo S, Lanfear J, Blomberg N (2021). ELIXIR: providing a sustainable infrastructure for life science data at European scale. Bioinformatics.

[15] Fortunato L, Galassi M (2021). The case for free and open source software in research and scholarship. Phil Trans R Soc A.

[16] R: a language and environment for statistical computing.

[17] Python.

[18] Wilkinson MD, Dumontier M, Aalbersberg IJJ (2016). The FAIR guiding principles for scientific data management and stewardship. Sci Data.

[19] Pressman RS (2010). Software Engineering: A Practitioner’s Approach.

[20] Bauch A, Adamczyk I, Buczek P (2011). openBIS: a flexible framework for managing and analyzing complex data in biology research. BMC Bioinformatics.

[21] hasanregius/workflow-based-information-management. GitHub.

[22] Rudolph K, Schubert M (2025). Box: write reusable, composable and modular R code. CRAN: Package box.

[23] Ben-Kiki O, Evans C, döt Net I (2009). YAML Ain’t Markup Language (YAMLTM) Version 12.

[24] (2025). Curl: a modern and flexible web client for R. CRAN: Package curl.

[25] stenevang/sftp. GitHub.

[26] (2025). RCurl: general network (HTTP/FTP/...) client interface for R. CRAN: Package RCurl.

[27] Di Germanio C, Deng X, Balasko B (2023). P‐CB‐3 | anti‐spike and nucleocapsid antibody dynamics following SARS‐CoV‐2 infection and vaccination: implications for sourcing COVID‐19 convalescent plasma. Transfusion.

[28] Grebe E, Stone M, Spencer BR (2024). Detection of nucleocapsid antibodies associated with primary SARS-CoV-2 infection in unvaccinated and vaccinated blood donors. Emerg Infect Dis.

[29] Stone M, Grebe E, Sulaeman H (2022). Evaluation of commercially available high-throughput SARS-CoV-2 serologic assays for serosurveillance and related applications. Emerg Infect Dis.

[30] Sulaeman H, Grebe E, Dave H, Chao DY (2023). Evaluation of Ortho VITROS and Roche Elecsys S and NC immunoassays for SARS-CoV-2 serosurveillance applications. Microbiol Spectr.

[31] Naeem M, Jamal T, Diaz-Martinez J, Pan JS, Balas VE, Chen CM (2022). Advances in Intelligent Data Analysis and Applications.

[32] Gupta A, Kumar S, Kumar A, Mandal S (2024). Big data in bioinformatics and computational biology: basic insights. Methods Mol Biol.

[33] Xia J, Wang J, Niu S (2020). Research challenges and opportunities for using big data in global change biology. Glob Chang Biol.

[34] Awotunde JB, Oluwabukonla S, Chakraborty C, Bhoi AK, Ajamu GJ, Hassan SA, Mohamed AW, Alnowibet KA (2022). Decision Sciences for COVID-19: Learning Through Case Studies.

[35] Nathan R, Monk CT, Arlinghaus R (2022). Big-data approaches lead to an increased understanding of the ecology of animal movement. Science.

[36] Glassman RB (1973). Persistence and loose coupling in living systems. Syst Res.

[37] Kasprzyk A (2011). BioMart: driving a paradigm change in biological data management. Database (Oxford).

